# The Efficacy of Brief School-Based Exercise Programs in Improving Pubertal Bone Mass and Physical Fitness: A Randomized Controlled Trial

**DOI:** 10.3390/ijerph18189648

**Published:** 2021-09-13

**Authors:** Xin Tian, Jian Fu, Jiaxue Tian, Yu Yang, Wenjie Liang, Wencui Fan, Renqing Zhao

**Affiliations:** College of Physical Education, Yangzhou University, Yangzhou 225002, China; tianxin96331@163.com (X.T.); Tianjiaxue0916@163.com (J.T.); YangYu922917@163.com (Y.Y.); liangwenjie1029@163.com (W.L.); fanw0123@163.com (W.F.)

**Keywords:** high-impact exercise, high-intensity interval training, bone mineral density, physical fitness, schoolchildren

## Abstract

Purpose: To examine the effects of three types of school-based exercises on bone health and physical fitness function in Chinese boys and girls. Methods: One hundred and seventy-four Chinese boys and girls were randomly assigned into four groups: (1) sham exercise (ShEx); (2) high-impact exercise (HiEx); (3) high-impact exercise with various directions (HiExVi); and (4) high-intensity interval exercise (HiInEx). Speed of sound (SOS) and physical fitness parameters were determined before and after six-month intervention. Results: At the end of six-month intervention, participants in all groups show an increment of SOS compared with the baseline (*p* < 0.05), and the changes were higher in HiEx (mean: 38.878 m/s, 95% CI: 32.885~44.872, *p* = 0.001) and HiExVi groups (49.317 m/s, 42.737~55.897, *p* < 0.001) compared with ShEx group (20.049 m/s, 13.751~26.346). Six-month exercise training generated a reduction of percent of body fat (PBF) and waist–hip ratio (WHR) (*p* < 0.05). The decrease of PBF was greater in HiExVi (−1.222%, −1.820~−0.624, *p* = 0.012) and HiInEx groups (−1.395%, −1.809~−0.982, *p* = 0.003), whereas the reduction of WHR was larger in HiEx (−0.026, −0.039~−0.014, *p* = 0.009), HiExVi (−0.024, −0.036~−0.012, *p* = 0.021), and HiInEx groups (−0.035, −0.046~−0.024, *p* < 0.001) compared with ShEx group. Balance function (BLF), vital capacity (VC), standing long jump (SLJ), and sit up (SU) increased in all intervention groups (*p* < 0.05). The BLF increased in HiEx (6.332 s, 4.136~8.528, *p* = 0.001), HiExVi (10.489 s, 8.934~12.045, *p* < 0.001), and HiInEx groups (9.103 s, 7.430~10.776, *p* < 0.001) showed a greater change than that of ShEx group (1.727 s, 0.684~2.770). The increment of VC (273.049 mL, 199.510~346.587, *p* < 0.001) and SU (2.537 times/min, 0.639~4.435, *p* = 0.017) was higher in HiInEx group, whereas the accrual in SLJ was larger in HiExVi (7.488 cm, 4.936~10.040, *p* = 0.007) compared with ShEx group (58.902 mL, 7.990~109.814; −0.463 times/min, −2.003~1.077; 1.488 cm, −0.654~3.630). Conclusion: The brief school-based exercises were effective in improving schoolchildren’s health, but they showed different effects, with HiEx mostly improving bone health, HiInEx largely benefiting physical fitness function, and HiExVi enhancing both bone and physical fitness.

## 1. Introduction

Puberty is the pivotal period for the growth and health of boys and girls. During this critical growth “window”, they experience the fastest growth velocity and acquire the major part of physiological and psychological functions, such as height increase, weight gain, bone mass accumulation, increment of cardiovascular capacity, development of nerve, immune, and endocrine systems [1,2]. Optimizing the growth and health of students is one of the essential goals of education in school, and increasing physical activities is one feasible strategy. 

However, physical inactivity, the term used to refer to inability to achieve the recommended levels of physical activity for health [3], is prevalent and affects health problems in many aspects [4], including the central nervous system, cardiopulmonary function, metabolism, muscle strength, bone mass, and immunity, which are considered to be the risk factors of obesity [5], cardiovascular disease [6], diabetes [7], and cancer [8], and it is recognized that risk factors for many chronic diseases usually have their origins in childhood and adolescence [9,10]. A recent survey of adolescents in 105 countries found that 14.9% of boys and 21.2% of girls never performed physical activity [11]. In China, the average time that adolescents spend on physical activity daily was about 38 min, much lower than that in Canada, the UK, the US, and most European countries [12]. A recent survey on Chinese school boys and girls showed that the rate of obesity was 20.6% and 12.8%, respectively [13], and Chinese Physical Education and Health Monitoring (CPEHM) reported the level of cardiorespiratory endurance, flexibility, coordination, and many other physical fitness functions in Chinese adolescents were lower than that of Japanese students with similar age [14]. 

Many factors cause adolescents to have less time to exercise. In China, boys and girls often have a relatively longer school time (about 10 h per day) with heavy homework (spending about 2.5 h per day), resulting in shorter exercise time and bedtime. Additionally, parents are willing to let students spend their leisure time learning rather than doing physical activities [15]. Meanwhile, physical inactivity was also prevalent among adolescents worldwide [16,17]. Though students of other countries may have less academic burden, they tend to not choose to do exercise rather than spending the time after school in sedentary behavior [18]. America National Survey of Children’s Health showed that only 32.9% of children have less than 2 h of screen time per day [19], and another study showed that the prevalence of excessive screen time in 12 European countries exceeded 90% [20]. Therefore, developing a brief exercise is an efficient and convenient strategy for improving the health of adolescents.

High-intensity interval exercise, which consists of intermittent bursts of efforts interspersed with short rest, has been recognized as a feasible and effective approach for improving adolescents’ cardiorespiratory fitness [21], muscle strength [22], body composition [22], etc. However, this exercise pattern may have some shortcomings in bone mass accumulation because the bone is more sensitive for mechanical loading [23,24]. High-impact exercise can increase bone density by exposing the bone to high-level mechanical strain [25]. It is reported that even if brief, shorter high impact exercise can improve bone mass [26,27] and geometry [28] in adolescents. Given that it is simple and requires less time, it may be a feasible intervention strategy for students. Additionally, it is reported that high-impact exercise with various directions can well challenge balance and posture control [29] and increase muscle strength [30]. Adolescents might get more benefits from this type of exercise. However, the efficacy of those intervention protocols on bone health and physical fitness function remains unclear, and the findings from this study might provide a feasible strategy for promoting adolescents’ health. Therefore, we conducted three different exercise interventions among Chinese students to test the hypothesis that brief, school-based exercises might benefit schoolchildren by improving physical fitness and bone health, and different exercise patterns probably generate differing effects. 

## 2. Method

### 2.1. Study Oversight

We conducted the Exercise for Health in Schoolchildren (EHSC) from September 2020 to March 2021 at Yangzhou city (ChiCTR2100049952). The study was approved by the institutional review board of Jinhu Hospital of Traditional Chinese Medicine (JHZYY202010). All participants provided written informed consent. All the authors had access to the data and vouch for the integrity, accuracy, and completeness of the data and analyses and for the fidelity of the study to the protocol. 

### 2.2. Participants

One hundred and eighty-nine volunteers were recruited from local middle schools. All students received comprehensive medical screening, and eligible participants should meet the following criteria: (1) between 11 and 15 years of age; (2) not obese (body-mass index (the weight in kilograms divided by the square of the height in meters) ≤ 30); (3) healthy, not having cardiovascular or respiratory diseases, musculoskeletal injuries (bones, muscles, tendons, ligaments, among others), history of major surgery, and mental disorder or neurological illness, and capable of conducting the exercise regimens. Fifteen persons who were overweight were excluded. A total of 174 schoolchildren were eligible for the study. Of these participants, 4 students missed the final tests, and 6 did not complete the training protocol. Eventually, only 164 schoolchildren (92 boys and 72 girls) were included in the data assessment.

### 2.3. Design

Randomization was conducted with a computer-generated allocation schedule through our research group, which was independent of the rest of the study staff. The allocation sequence was concealed from the research assistant enrolling and assessing participants. Subjects were randomly allocated into four groups: (1) sham exercise/control (ShEx) (n = 43), only doing light stretching exercise assumed not to affect bone metabolism and physical fitness [31]; (2) high-impact exercise (HiEx) (n = 44), jumping vertically 12 beats per minutes with a height of at least 30 center meters, with two 3 min jumping exercises followed by a 4 min break. A metronome and cones were respectively used to control the interval and height of each jump; (3) high-impact exercise with various directions (HiExVi) (n = 43), jumping as that of HiEx group but changing directions after rising and landing at different points, such as the front, left, right, and initial point in the sequence; (4) high-intensity interval exercise (HiInEx) (n = 44). The training program consisted of six 1 min mountain climbing exercises at 77–95% HR_max_ interspersed with 30 s recovery periods. And the first bout started after a 1 min warm-up which was used to reach the target heart rate. The optical sensors (OH1, Polar Elector Malaysia Sdn Bnd, Helsinki, Finland) were used to adjust the magnitude of load during the exercise intervention. All participants received 10 min exercise intervention during lunch break three times per week for six months. The exercise intervention was conducted in a school health center and supervised by a physical education teacher who was not involved in any other aspect of the study. The times of participation and injuries during training were recorded.

### 2.4. Anthropometry

The body height and weight of participants were determined using a height and weight measuring instrument (HK6800-ST, Hengkang Inc, Guangdong, China). The accuracy for body height and weight measures were set at 0.1 cm and 0.1 kg, respectively. Participants wore tight-fitting clothes and were barefoot during the test. Body mass index (BMI) was calculated by dividing body weight by height squared (kg/m^2^).

### 2.5. Extracurricular Physical Activity

Daily extracurricular exercise log was recorded, in which the participants described the type, duration, intensity, and frequency of every extracurricular activity. Each type of physical activity was assigned a MET score on the basis of its energy cost [32], and physical activity-related energy expenditure (MET·h/week) was computed as the summed product of frequency, duration, and intensity in a week.

### 2.6. Total Energy and Calcium Intake

A combination of bookkeeping and weighing methods was used to collect information about energy and calcium intake. All participants recorded the category and weight of food intake at any time during one week under the requirements of the “Daily Diet Log” [33]. It was assigned on a daily basis from Monday to Friday before school was over and collected after attending school on the third day except for the weekend (the Saturday and Sunday logs distributed on Friday were collected on the following Monday).

The lunch and dinner the students ate at school had a fixed menu and uniform tableware, and investigators measured the weight of the dish with side dishes and condiments. Meanwhile, we distributed an electronic food scale to every family and required parents to help participants to record their diet outside the school. Additionally, each group had two investigators responsible for providing food record guidance both online and offline. Before the formal investigation, we also had conducted a two-day pre-investigation to figure out potential problems in the process. Energy and calcium intake in the diet was calculated by “China Food Composition Table (2019)”.

### 2.7. BMD Measure

Quantitative ultrasound (CM-100, Furuno Inc, Hyogo, Japan) was used to measure the BMD [34,35]. After ultrasound coupling gel was applied on the sensor head and the left calcaneus, participants put the foot into the instrument barefoot. When the lower heel closed contact with the instrument, the operator fixed the position then started the measurement. The measurement result was printed by the printer in the instrument include the speed of sound (SOS) and T-value.

### 2.8. Physical Fitness Measure

Physical fitness testing of National Course Standard of Physical Education and Health [36] includes body composition (such as body height, weight, body fat), lower limb muscular strength (standing long jump), muscle endurance (1-min sit-up), cardiorespiratory endurance (vital capacity), and balance (closed-eye foot balance).

Body fat percentage (PBF) was measured by a human body composition analyzer (Inbody770, InBody Co., Seoul, Korea) [37], and the ratio of the upper edge of the navel and the circumference of the most prominent part of the gluteus was measured by a non-stretching measuring tape (the value was accurate to 0.1 cm) determined the waist-to-hip ratio (WHR).

The standing long jump measured the horizontal jump distance of each person. Everyone jumped twice, and the better result was recorded. The sit-up test calculated the maximum number of repetitions in a minute.

Vital capacity was assessed by the FHL-II spirometer (Xindong Huateng Inc, Beijing, China) [38]. After the first maximum exhalation, participants rested for a minute before the second test, and the best performance was recorded.

Balance ability was assessed by the duration of one-leg standing with eyes closed [39,40]. Before the test started, the participant stood on flat ground and gazed at the horizontal marking line one meter away with hands on the hip. After receiving the instruction to start, the participant lifted right foot to the left ankle joint position and closed eyes at the same time. The test was ended with the following conditions of participants: (1) eyes open, (2) the raised foot touches the supporting leg, (3) the supporting leg moves, and (4) the hand leaves the hip.

### 2.9. Statistics

SPSS 20.0 (SPSS Inc., Chicago, IL, USA) was used to analyze the data. Descriptive statistics are presented as mean ± standard deviation (SD). Shapiro–Wilk test and Levene’s test were used for examining the normal distribution of the data and homogeneity of variance (*p* > 0.05 for normal distribution or homogeneity of variance). A two-way repeated-measures ANOVA was used to compare the outcomes of BMD and physical fitness parameters across the different conditions (group and time). If the results were significant, paired *t*-tests were used to analyze the changes from pre-intervention to post-intervention. One-way ANOVA and post hoc Bonferroni were used to analyze the difference in change magnitude between groups. *p* < 0.05 was considered significant.

## 3. Result

### 3.1. Baseline Characteristics of Participants

Participants’ characteristics are listed in Table 1. Briefly, the age, body height, body weight, body mass index, and other characteristics were not different between the four groups (Table 1).

### 3.2. Changes in SOS Evaluation

**Figure 1:**
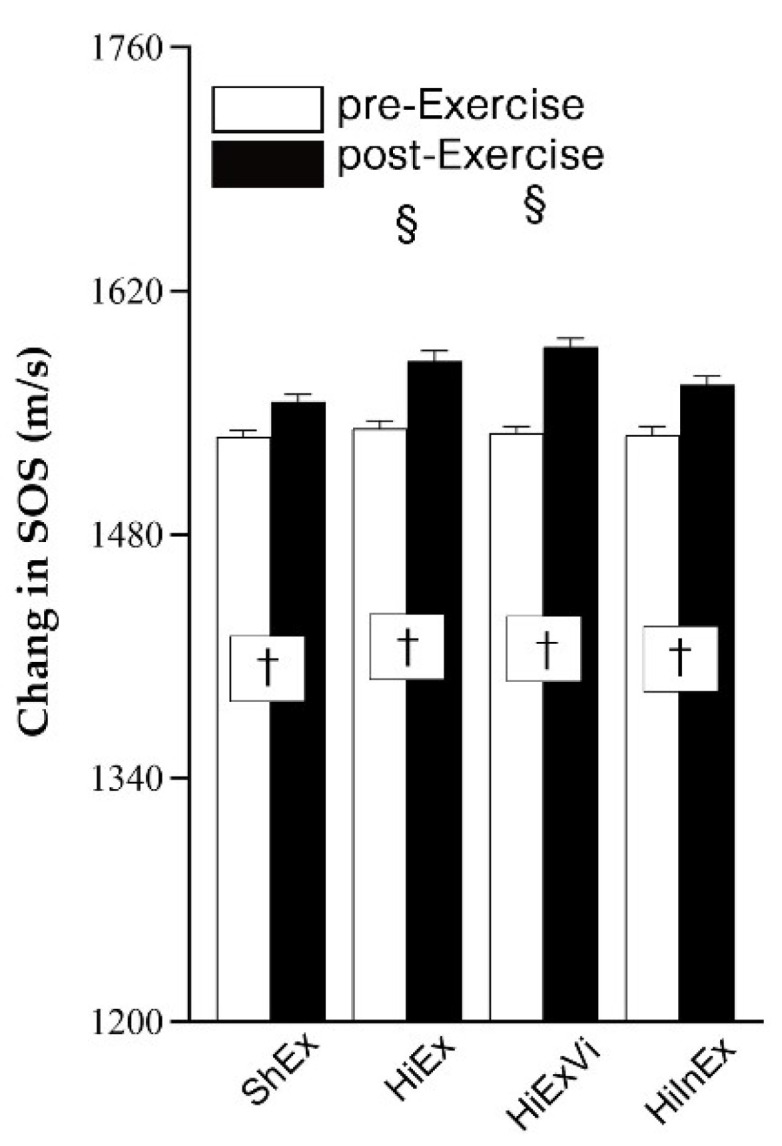
The change in speed of sound (SOS). ShEx—sham exercise; HiEx—high-impact exercise; HiExVi—high-impact exercise with various directions; HiInEx—high-intensity interval exercise. Data are presented as mean ± standard deviation (SD). ^†^ indicates that the difference between pre- and post-exercise is significant (*p* < 0.05). ^§^ denotes that the difference in change magnitude is significant in HiEx, HiExVi, and HiInEx groups compared with ShEx group (*p* < 0.05).

The baseline SOS was similar between groups (*p* = 0.891). A two-way repeated-measures ANOVA demonstrated that the time, group, and interaction effect were all significant (Table 2). Six-month intervention generated a greater increment of SOS compared with baseline measurement in all the four groups (Figure 1).

One-way ANOVA revealed that the changes in SOS between baseline and six-month intervention were higher in HiEx (mean: 38.878 m/s, 95%CI: 32.885~44.872, *p* = 0.001) and HiExVi groups (49.317 m/s, 42.737~55.897, *p* < 0.001) compared with ShEx group (20.049 m/s, 13.751~26.346). Alhough HiInEx intervention (28.902 m/s, 20.393~37.412 *p* > 0.05) generated a relative higher increment of SOS compared with ShEx, the difference was not significant (Figure 1).

### 3.3. Physical Fitness Evaluation

The baseline physical fitness parameters were similar between groups (*p* < 0.05). Additionally, all physical fitness indicators showed a significant difference in time and interaction effect (Table 2).

**Figure 2:**
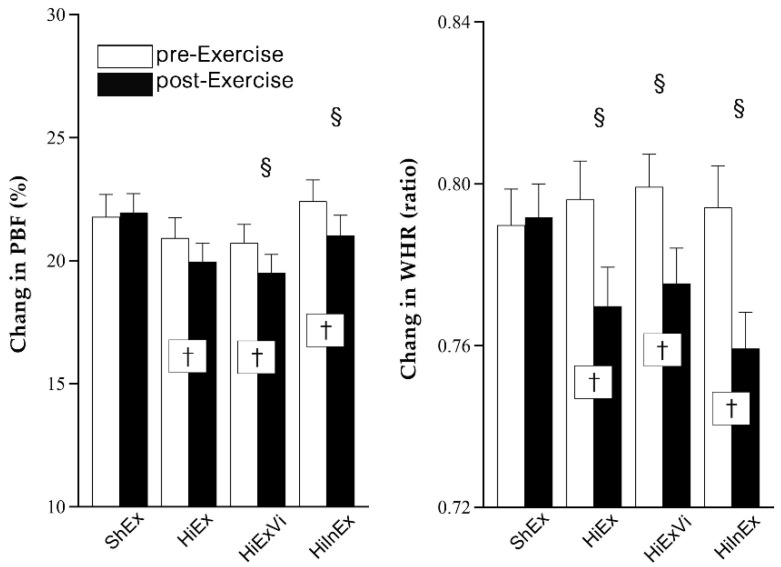
The change in percent of body fat (PBF) and waist–hip ratio (WHR). ShEx—sham exercise; HiEx—high-impact exercise; HiExVi—high-impact exercise with various directions; HiInEx—high-intensity interval exercise. Data are presented as mean ± standard deviation (SD). ^†^ indicates that the difference between pre- and post-exercise is significant (*p* < 0.05). ^§^ denotes that the difference in change magnitude is significant in HiEx, HiExVi, and HiInEx groups compared with ShEx group (*p* < 0.05).

Exercise intervention induced remarkable reduction of PBF and WHR compared with baseline (*p* < 0.05). The change of PBF was greater in HiExVi (−1.222%, −1.820~−0.624, *p* = 0.012) and HiInEx groups (−1.395%, −1.809~−0.982, *p* = 0.003) compared with ShEx group (0.183%, −0.593~0.959). Additionally, the reduction of WHR was larger in HiEx (−0.026, −0.039~−0.014, *p* = 0.009), HiExVi (−0.024, −0.036~−0.012, *p* = 0.021), and HiInEx (−0.035, −0.046~−0.024, *p* < 0.001) groups than ShEx group (0.002, −0.012~0.016) (Figure 2).

**Figure 3:**
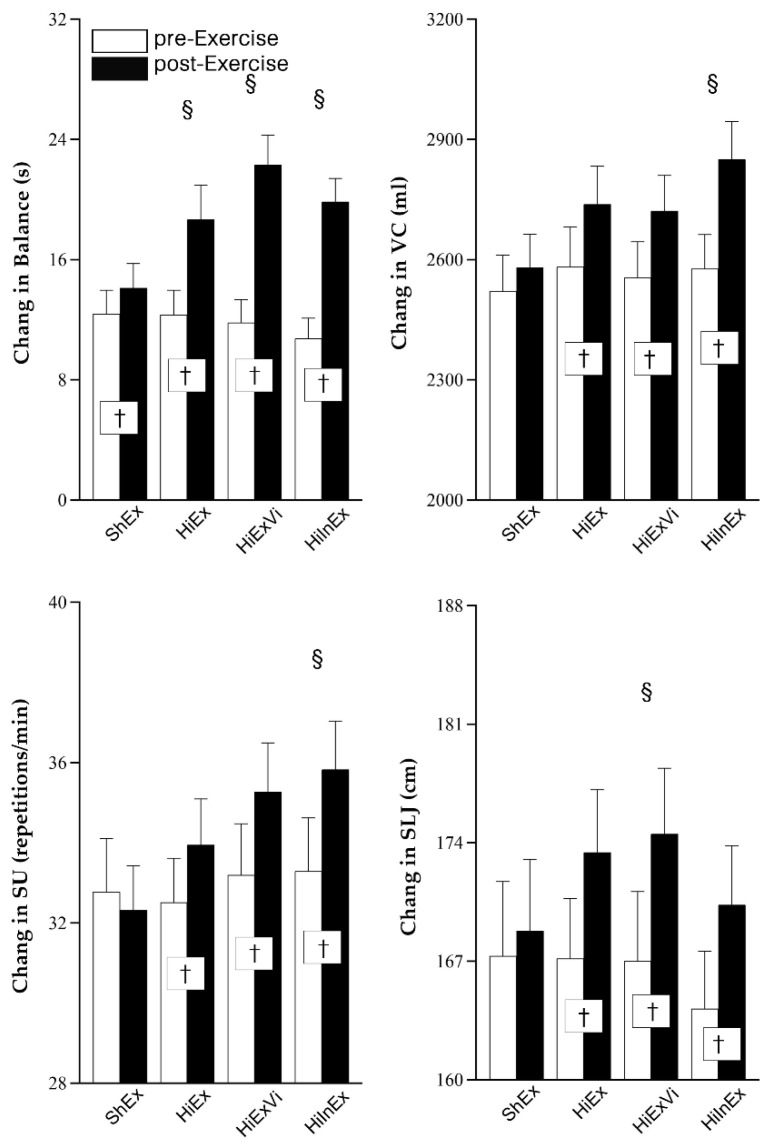
The change in balance function, sit up (SU), vital capacity (VC), and standing long jump (SLJ). ShEx—sham exercise; HiEx—high-impact exercise; HiExVi—high-impact exercise with various directions; HiInEx—high-intensity interval exercise. Data are presented as mean ± standard deviation (SD). ^†^ indicates that the difference between pre- and post-exercise is significant (*p* < 0.05). ^§^ denotes that the difference in change magnitude is significant in HiEx, HiExVi, and HiInEx groups compared with ShEx group (*p* < 0.05).

At the end of six-month exercise intervention, the balance function was improved in all groups (Figure 3), and increment was higher in HiEx (6.332 s, 4.136~8.528, *p* = 0.001), HiExVi (10.489 s, 8.934~12.045, *p* < 0.001), and HiInEx groups (9.103 s, 7.430~10.776, *p* < 0.001) than ShEx group (1.727 s, 0.684~2.770).

Vital capacity (VC), sit up (SU), and standing long jump (SLJ) increased in all exercise groups except for the ShEx group at the end of the six-month intervention (Figure 3). The increments of VC (273.049 mL, 199.510~346.587, *p* < 0.001) and SU (2.537 times/min, 0.639~4.435, *p* = 0.017) were higher for HiInEx group than ShEx group (58.902 mL, 7.990~109.814; −0.463 times/min, −2.003~1.077), whereas the accrual in SLJ was larger in HiExVi (7.488 cm, 4.936~10.040, *p* = 0.007) compared with ShEx group (1.488 cm, −0.654~3.630).

## 4. Discussion

This is the first study to simultaneously explore the effects of HiEx, HiExVi, and HiInEx on the BMD and physical fitness of school boys and girls. Our study proved brief school-based exercises to be effective in improving schoolchildren’s health; even merely 10 min physical activities could generate positive outcomes. However, the three exercise patterns benefited different aspects of bone and physical fitness.

According to the mechanostat theory, bones adjust their strength and content to adapt to external physiological loads [41]. Previous studies [42,43] suggested that high-level strain produced by high magnitude loads, such as during high-impact exercise, can promote the formation of bone periosteum and improve the bone geometry and strength index. Petit et al. reported that the 7-month high jump intervention significantly increased the cortical thickness of the femoral neck among girls in early puberty [44]. Other studies showed that 20 countermovement jumps enhanced the bone mass and cross-sectional area of multiple weight-bearing sites of adolescents [45,46]. Our findings are consistent with the results of previous studies. The osteogenic effect depends on the internal muscle activity and the impact of external force [47], which regulate the proliferation and maturation of osteoblasts and osteoclasts through the signal transduction system of bone cells and improve the positive balance of bone metabolism. High-impact load programs can produce the two types of osteogenic forces and generate beneficial effects on bone mass and structure [48]. Our findings further demonstrated that the regularly brief HiEx program promoted bone health among schoolchildren. As a matter of fact, with the increasing of stimulus durations, the bone cells gradually become insensitive to the mechanical load [49]. Umemura et al. showed that there was no significant difference in bone mass changes between jumping 100 times per day and jumping 40 times per day [50]. Appropriate rest between successive loading cycles could produce a greater bone response than no rest [51].

Interestingly, the HiExVi program showed a greater gain of BMD compared with HiEx. Odd-impact exercise frequently generates an osteogenic effect on bone, such as that of high-impact exercise [52]. Moreover, movement in various directions can provide unusual stimuli such as compression, bending, twisting, etc., and distribute the stress to the multiple planes of the weight-bearing part [53]. The amplification of BMD increment results from larger strain peaks and irregular loads during exercise [54]. The bones that receive multi-directional loads and high ground reaction forces at the same time are stiffer and more fracture-resistant [55]. Additionally, changing directions increases the additional challenge of balance control which generates more muscle tasks [56]. When the skeletal muscles resist or promote joint movement with higher contractile force, the bone load increases accordingly [57].

Jumping could store and release elastic energy to generate larger muscle recruitment and improve muscle strength and explosive power [58]. Moreover, odd high-impact exercise includes high acceleration and deceleration forces from various directions, and these forces usually lead to an increase in muscle strength. The increase of standing long jump performance in the HiExVi group represented the elevated muscle explosive force and improved neuromuscular function and sensorimotor control [56]. Jumping at a relatively high velocity could benefit bone health and neuromuscular activation, but it leads to lower extremity movement as well, which generates greater load on the anterior cruciate ligament and increases the possibility of injuries [59].

Balance ability is related to lower limb muscle strength, which can compensate and correct for the sway without losing balance [60]. Improvement in closed-eye foot balance achievement is a combined outcome of many factors, such as muscle strength, reaction time, vestibular function, peripheral sensation, and posture adjustment [61]. Both high-impact and high-intensity exercise interventions can bring better challenges to balance control and stimulus adaptation and then provide benefits for postural stability both in anteroposterior and mediolateral [56].

Exercises that continuously increase heart rate and energy expenditure are understood to help weight control and cardiovascular health [62]. HiInEx can induce the same effect as continuous moderate-intensity exercise, sometimes even better [63]. Trapp et al. reported that HiInEx only needed 50% of the time to achieve the same energy consumption as steady-state exercise and reduced body fat by 2.5 kg after a 15-week intervention [64]. Other studies further demonstrated that eight weeks of the HiInEx program reduced abdominal fat by 0.13 kg [65], and the loss of abdominal fat mass was not different between males and females [66]. One explanation for the fat loss induced by HiInEx is that high-intensity training can result in the improvement of serum adiponectin concentration [67,68]. In fact, it is well known that there is an inverse relationship between circulating adiponectin level and obesity, especially visceral fat accumulation [69]. The brief school-based HiInEx program can improve body composition, but this effect is the benefit of long-term exercise intervention. On the contrary, HiInEx lasting for less than four weeks did not yield a clinically significant reduction in body fat [70]. HiExIv could also reduce body fat, but it is unknown whether this effect is correlated to the change in adiponectin level.

Some people assume that increasing the time of low-intensity exercise can also enhance cardiorespiratory function like that of high-intensity exercise. However, even if the amount of exercise is doubled, the improvement of cardiorespiratory function still cannot reach the gains of high-intensity exercise [71]. High-intensity exercise can induce a series of physiological adaptations, including the improvement of mitochondrial content and peripheral vascular structure and function [72], and it seems to be a key factor in promoting cardiorespiratory capacity [73]. Additionally, Schmidt et al. reported that high-intensity circuit training, using body weight as resistance, has positive effects on enhancing muscle endurance, even if it is performed for 7 min only [74]. Our findings were in agreement with previous studies.

Although HiInEx proved effective in improving physical fitness [75], its effect on bone health among school boys and girls remains unclear [76]. Our study suggested that the BMD of the HiInEx group increased compared with the baseline, but the change was not significant compared with that of the ShEx group. This indicated that the increment of BMD in the HiInEx group might result from age accrual but not the effects of HiInEx intervention. According to mechanotransduction, bone tissue perceives mechanical force through fluid flow within the bone matrix [77]. Adding a rest between two movements causes fluid shift in one direction during each load application and flow in the reverse direction when the load disappears and then generate periodic stress stimulation [78]. Reaching high intensity in a short time inevitably needs to shorten the movement interval, and therefore it does not have enough time for driving fluid flow from one direction to another. High intensity represents high repetitions and shortened intervals, which weaken bone cell adaption in response to mechanical stimuli [79].

### Limitation

Our research has some limitations. Considering a relatively large sample size and participant opinions, we only used non-radiation quantitative ultrasound (QUS) to measure BMD and did not analyze the changes in bone mass, bone structure, bone strength, and other bone parameters. However, as QUS is proven to have a good correlation with BMD results measured by dual-energy X-ray absorptiometry (DXA) [80,81] and quantitative computed tomography (QCT) [82], our results have clinical potential. Moreover, due to the advantages of this measure, e.g., safe, convenient, and low-cost, it could be widely applied for large population health surveys; for example, T score based upon QUS measures has been used for bone health surveys among children and adolescents [83] and screening osteoporosis among adults in China [84]. Another limitation was that we used one-leg standing test to measure balance function rather than examining COP (center of the pressure), a better method for balance assessment. However, one-leg standing test is also a reliable method for examination of balance function [39,40] and can easily be applied in large population surveys, especially in developing countries.

## 5. Conclusions

Our findings provide population-based evidence on the effectiveness of brief school-based exercises in improving schoolchildren’s health. Differing programs showed different effects on bone health and physical fitness, with HiEx mostly improving bone health, HiInEx largely benefiting physical fitness function, and HiExVi enhancing both bone and physical fitness. However, it should be kept in mind that physical education school discipline is more complex to achieve physical, cognitive, and emotional benefits. Therefore, although the benefits noticed in the present study are related to morphological and physical dimensions, the present results should not be decisive to define the minimal time for physical education stimulus. For detecting the benefits of the three types of exercise among school boys and girls, further population-based studies are still needed to measure the changes of other parameters.

## Figures and Tables

**Table 1:** Characteristics of participants between groups (mean ± SD).

	ShEx	HiEx	HiExVi	HiInEx	*p*-Value
Age (year)	12.3 ± 0.5	12.3 ± 0.5	12.3 ± 0.5	12.2 ± 0.4	0.763
Height (cm)	163.7 ± 8.2	163.1 ± 6.7	164.2 ± 7.3	163.8 ± 8.0	0.926
Weight (kg)	53.3 ± 11.1	52.9 ± 9.9	52.7 ± 9.5	54.0 ± 14.3	0.954
BMI (kg/m2)	19.8 ± 3.6	19.8 ± 2.9	19.4 ± 2.4	19.9 ± 4.1	0.919
Calcium Intake (mg/week)	2340 ± 566	2409 ± 724	2374 ± 803	2336 ± 729	0.963
Energy Intake (kcal/day)	1310 ± 161	1352 ± 195	1433 ± 167	1335 ± 292	0.053
Extracurricular Exercise (MET-Hours/week)	11.5 ± 11.5	11.0 ± 12.3	10.4 ± 14.1	11.7 ± 14.9	0.983

Notes: HiEx—high-impact exercise; HiExVi—high-impact exercise with various directions; HiInEx—high-intensity interval exercise.

**Table 2:** The changes between and within group.

	F (hp2)		
	Time Effect	Time × Group Interaction	Group Effect
SOS	401.670 (0.715) *	13.630 (0.204) *	2.866 (0.051) *
PBF	28.772 (0.152) *	5.052 (0.087) *	1.241 (0.023)
WHR	45.561 (0.222) *	6.640 (0.111) *	0.499 (0.009)
Balance	280.660 (0.637) *	21.935 (0.291) *	0.871 (0.016)
VC	80.760 (0.335) *	5.794 (0.098) *	0.579 (0.011)
SLJ	69.212 (0.302) *	4.228 (0.073) *	0.199 (0.004)
SU	15.873 (0.090) *	3.540 (0.062) *	0.624 (0.012)

Notes: Analysis by a two-way repeated-measures ANOVA, * indicates *p* < 0.05. SOS—speed of sound; PBF—percent of body fat; WHR—waist–hip ratio; SU—sit up; VC—vital capacity; SLJ—standing long jump.

## Data Availability

The datasets used and/or analyzed during the current study are available from the corresponding author on reasonable request.
